# Prescribing Behavior of General Practitioners for Generic Drugs

**DOI:** 10.3390/ijerph17165919

**Published:** 2020-08-14

**Authors:** Berna Tuncay, Sergio Pagano, Mario De Santis, Pierpaolo Cavallo

**Affiliations:** 1 Department of Economics, Koc University, 34450 Istanbul, Turkey; betuncay@ku.edu.tr; 2 Department of Physics, University of Salerno, 84084 Salerno, Italy; spagano@unisa.it; 3 Consorzio Mega Ellas, 84132 Salerno, Italy; mariodesantis@osservatoriosanitario.it; 4 Istituto Sistemi Complessi del Consiglio Nazionale delle Ricerche (ISC-CNR), 00185 Rome, Italy

**Keywords:** generic drugs, general practitioner, prescribing behavior, electronic health records

## Abstract

The factors influencing General Practitioners’ (GPs) prescribing behavior are diverse in terms of health care policies and regulations, GPs’ education and experience, demographic trends and disease profiles. Thus, it can be useful to analyze the specific local patterns, as they affect the quality of healthcare and the stability of the healthcare market. The aim of the present longitudinal retrospective study is to investigate the prescription of generic drugs in a database of about 4.6 million prescriptions from a sample of 38 GPs practicing in Salerno, Italy, within a timeframe of 15 years, from 2001 to 2015. The GPs in our study show a general tendency to increase prescriptions of generic drugs during the studied time span, to fulfill regulatory obligations and with some differences in prescription behavior according to age, gender and experience. The generics prescription depends also on the different diagnoses, with some diagnostic areas showing a greater generic drug prescription rate. Expanding this research to larger datasets would allow deepening the knowledge of the patterns of GPs’ prescribing decisions, to provide evidence to be used in comparison between different national settings.

## 1. Introduction

Significant differences exist among the prescribing behavior of GPs for brand-name and generic drugs in the major European countries [1,2,3]; these may create problems because of adverse effects on the cost of healthcare and, therefore, on the local economy.

During the 1990s, Italian health authorities identified prescribing patterns in general practice as a particular area for improvement in the healthcare industry, because healthcare expenditure was growing out of control. Since that time, academic research on the prescribing behavior of GPs in Italy has remained very limited; therefore, it is difficult to identify patterns and behaviors and, more importantly, how they are affecting patient health and local economies. A review [4] performed on the generic drug experience in eight countries underlined the presence of barriers, including negative perceptions, lack of coherent policies and other factors, with different lessons learned.

Several researchers have proposed models attempting to explain the process of decision making that leads a GP to prescribe a particular drug over another [5,6], that is, a brand-name drug versus a generic.

Some of these models indicate external factors related to GPs’ prescribing patterns such as the introduction of new pharmaceutical policies, marketing and promotional agendas developed by pharmaceutical companies, the introduction of new, effective drugs and trade-name drugs and the less expensive generic substitutes for these drugs.

Other factors are based on the cognitive processes that influence GPs’ prescribing decisions. For instance, pharmaceutical characteristics related to the treatment such as drug effects, side-effects, co-morbidity conditions and conceptual factors such as past experiences of the patient or patient preferences and habits are important factors affecting GPs’ prescribing behaviors [7]. Further, GP gender [8] is another determinant that can influence the prescribing decisions.

Despite these factors and their effects on decisions, it has been widely accepted that once a GP’s prescribing behavior is established, this pattern is not easily altered [9]. As generic drugs enter the market, some GPs change their personal lists to account for them; however, most do not. The reasons behind these decisions are not clear or consistent; for instance, Lambert et al., state that “prescribing behavior might be a function of patient-specific characteristics” [10].

In any case, there are occasions in which branded prescribing is appropriate, when there are, for instance, drugs with a narrow therapeutic index, or certain modified- or controlled-release drugs that must necessarily be used, administration devices, biological drugs including biosimilars, multiple ingredient products, drugs with different licensed indications, or even drugs to ensure adherence to long-term medications, where differences in appearance between manufacturer’s products might cause confusion and anxiety.

The Italian drug prescription system includes the ATC [11] coding (Anatomical Therapeutic Classification), developed by the World Health Organization (WHO), which includes fourteen main anatomical/pharmacological groups, 1st level, each of which is further divided into either pharmacological or therapeutic, 2nd level, then chemical, pharmacological or therapeutic, 3rd and 4th level, and finally the chemical substance, 5th level.

Italy also uses a proprietary classification system [12], called AIC, “Autorizzazione Immissione Commercio” (that is, Marketing Authorization). The AIC classification includes the producer name, the drug name and the packaging. For instance, the Nimesulide, with ATC code M01AX17, is sold under a large number of AIC codes, many of which are generic.

Moreover, the regulatory system requests that for each prescribed drug, there must be the indication of the disease for which it is prescribed. The diagnosis is coded according to the International Classification of Diseases, Ninth Revision, Clinical Modification (ICD-9-CM) system [13]. It consists of a tabular list containing a numerical list of the disease code numbers, grouped into categories based on clinical typology, e.g., “diseases of the circulatory system”.

In general terms, a regulatory obligation was introduced, with the Italian law 405/2001, for the pharmacist to deliver the generic drug to the patient, unless the GP certifies that the branded drug is not replaceable for that patient. Furthermore, the Italian law 221/2012 also imposed to the GP the obligation to indicate the active principle in the prescription. However, the patient can always request to obtain the branded drug prescribed, and pay “out of pocket”, which is the difference between the preferred branded drug and the generic to which the offer is limited.

Given this knowledge, we hypothesize that the analysis of prescription databases can be a useful source of information to obtain specific data about generic vs. branded prescription.

The goal of our study is to provide valuable information about the potential reasons of prescribing generic or branded drugs by GPs, in terms of available parameters such as age, gender and professional experience and to suggest possible perspectives and methods for further research.

The paper is organized as follows. Section 2 provides information on data and empirical methodology. Empirical results are given in Section 3. Section 4 summarizes discussions. Finally, concluding remarks are given in Section 5.

## 2. Data and Methodology

We began our research by collecting prescriptions written by a population sample of 38 GPs operating in the area of the local health authority of Salerno, Italy. Most of them were active during the years for which the data were analyzed and were selected between the members of the GP Consortium that made the data for the present study available. These prescriptions represent about 70,000 patients, of whom about 57,000 visited the GPs in our sample within a time frame of 15 years, resulting in a total of about 4.6 million prescriptions. The prescription data are mainly used for administrative purposes by the National Health Organization to monitor and control expenses and to contain data on the prescribed items and limited information on patients and GPs. Although our study is limited to the Salerno area in Italy, it is important to underline that these administrative data are collected on a national level (a practice that many other nations follow as well) and, in most cases, represent a mostly unexplored source of information on the health status of the general population, in particular when their illnesses are not so severe to require hospitalization.

The prescriptions are extracted from the electronic health records (EHR) that are normally logged by GPs to account for patient visits and inserted into a database from which all the inferences and analyses have been performed.

The data contain information on the type of prescription (drug, test, referral, etc.), name and quantity of the prescribed drug, prescription date and type of cost exemption. To ensure full respect of patients’ and GPs’ privacy, all references to the patient’s identity and address are removed, and only age and gender characteristics are retained. This study has received permission from the relevant Ethics Committee, n. 59, released on 8 June 2016.

The statistical analysis was performed using the R Statistics Environment, using the DataTable package and the StatsDirect Software, providing the descriptive statistics (average, standard deviation and confidence limits), graphical analysis (boxplot) and analytic methods for comparison and correlation (Student’s *t*-test). Statistical significance is considered relevant for *p* < 0.05, and high significance is considered relevant for *p* < 0.01.

We conducted our analysis using a timeframe of about 15 years, from 31 December 2001 to 31 July 2015. During this time, the patients requested 2,778,766 visits that produced 4,614,159 drug prescriptions. The drug prescriptions are classified according to their ATC and AIC codes, which are embedded in the EHR system of all the Italian GPs.

Age distribution among the patients in our study is rather homogeneous among GPs. For instance, the average age of patients belonging to different GPs has a standard deviation of 2.7 and 3.2 years, for male and female patients, respectively. The 38 GPs consist of 29 males and 9 females and have an average age of 61.5 and 57.8 years, respectively. According to Italian regulations, each GP can assist a maximum of 1500 patients. The average number of patients per GP in our sample is about 1400. The prescribed drugs are defined branded or generic by examining their retail price, comparing it with the reference price data fixed by the Italian Health Authority, and checking for the presence of their AIC code [12] in the generic drugs list. Using this standard, we were able to qualify a total of 2,688,388 drug prescriptions, among which 409,773 were determined to be generic drugs.

## 3. Empirical Results

**Figure 1:**
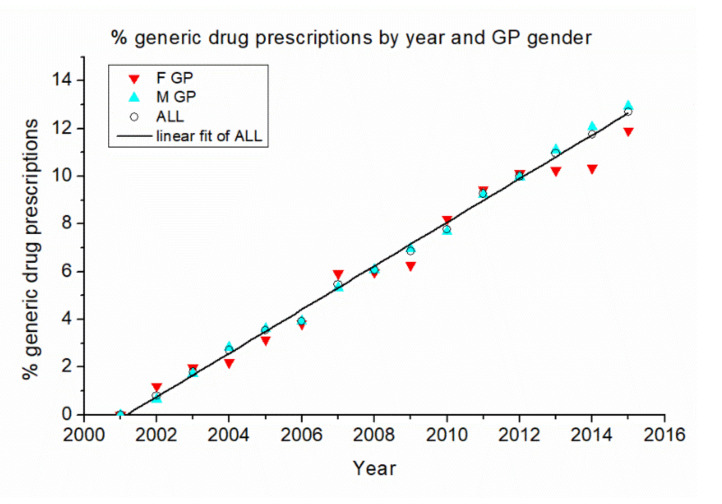
Percentage of generic drug prescriptions by year and GP gender. The black line is a linear fit of data referred to all GPs.

In Figure 1, we report the percentage of generic drug prescriptions, with respect to total drug prescriptions, by year and GP gender.

The data show a distinct linear increase over time. Although there is some variability in the case of female GPs, the total tendency closely follows a linear trend of 0.915 ± 0.013% per year, with a correlation coefficient r = 0.998 and *p* < 0.001. This indicates a constant tendency of GPs to adopt the national directives about the prescription of generic drugs and a parallel increase in the availability of generic alternatives to the other drugs.

When the GPs are analyzed individually, a greater variability emerges in their generic drug prescription habits.

**Figure 2:**
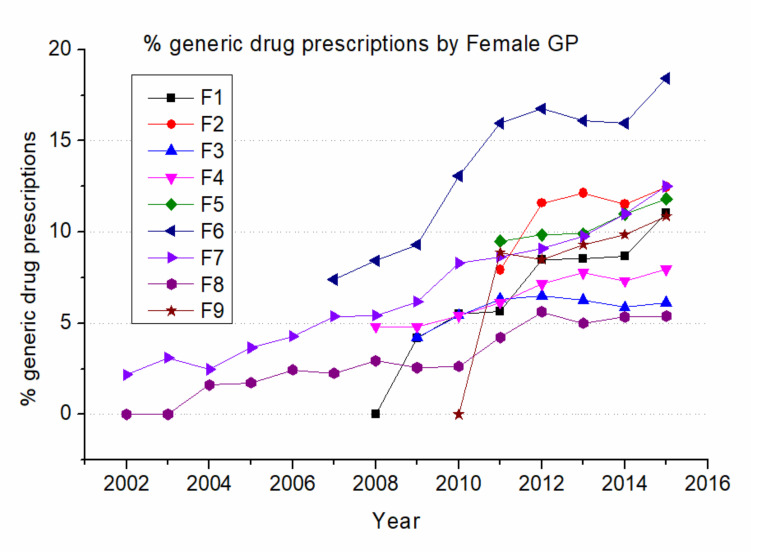
Percentage of generics over total drug prescriptions by female GPs. Female GPs are indicated with letters from F1 to F9.

**Figure 3:**
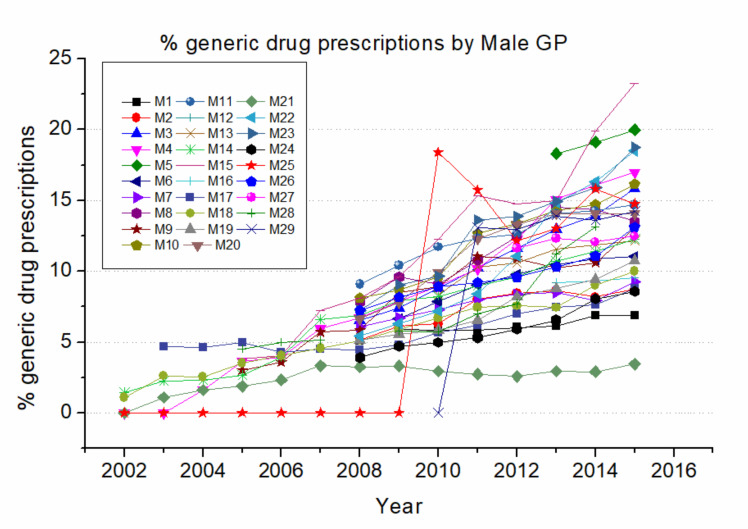
Percentage of generics over total drug prescriptions by male GPs. Male GPs are indicated with letters from M1 to M29.

Figure 2 shows the percentage of generic drug prescriptions year by year for female GPs (F GP), and Figure 3 shows the same for male GPs (M GP). Each curve covers the time span of the years of effective presence of GPs’ electronic records in the associated database.

In Figure 2, it is clearly shown how the individual F GP’s prescribing behavior has a lot of variability, with a range of values among different GPs spanning largely. Aside from a few cases of later adoption (F8 in the years 2002 and 2003, F1 in the year 2008, and F9 in the year 2010), all F GPs show a general tendency to increase their prescription of generic drugs during the time period, almost at the same rate. A notable observation is F7 who has a significantly higher tendency to prescribe generic drugs, indicating that the choice of a generic substitute is mostly a GP’s decision, rather than an externally determined action (i.e., the availability of generics).

Figure 3, when compared with Figure 2, shows a similar tendency, indicating that prescription of generics was nearly absent at the beginning of the time span studied, with a similar trend between different GP genders. A notable exception is the late adopter M25, who started to prescribe generics in 2009 but then quickly converged toward the values of all other GPs. Also worth noting is that M21 was an early adopter but consistently prescribed much less generic drugs compared to his colleagues. These cases again underline the large individual variability in the GPs’ prescription habits.

**Figure 4:**
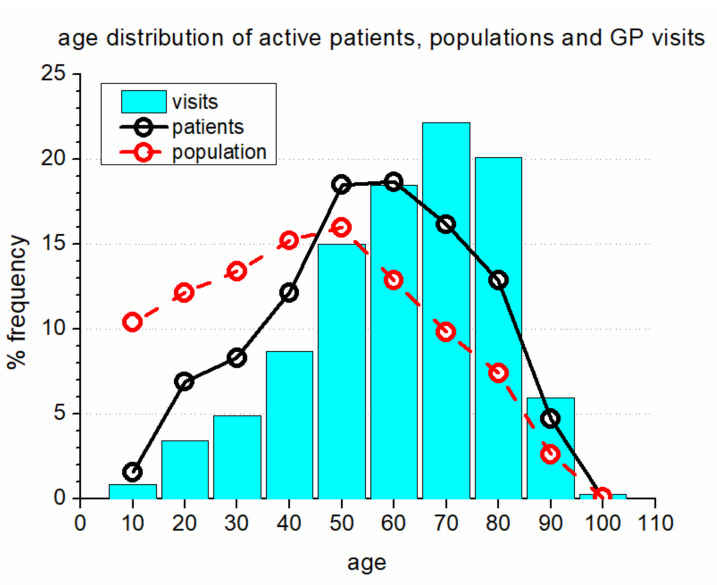
Age distribution of active patients, compared with that of general population and with the frequency of GP visits. The age groups are indicated by the mid value. The age range is ± 5 years.

Figure 4 presents the graph of the age distribution of “active” patients, who made at least one visit in 2015, versus general population.

In Figure 4, solid lines represent the age distribution of the active patients in our sample population, including 29,400 inhabitants, while dashed lines represent the age distribution of the general population of the Salerno province, consisting of 1,057,000 inhabitants. The vertical bars on the graph show the frequency of visits to GPs according to patients’ age range. In addition to a very small value for patients in the age range of 5–15 (because this population largely visits pediatricians), there is a clearly lower frequency of visits to GPs in patients below the age of 50 and a higher frequency of visits to GPs in patients above the age of 50.

Table 1 and Table 2 report the generic drug prescription data for female and male GPs respectively. In each table, there is an ID number for the GP, the age at the moment of the data sampling, the average number of generics prescribed per year (ANGP), calculated excluding the years with zero prescriptions, and the number of years (Gen Yrs) in which the GP has prescribed generics.

The descriptive statistics for the data of Table 1 and Table 2 are shown in Table 3. The data in Table 3 were compared using one-way ANOVA and Student’s unpaired *t*-test, showing that the only significant difference was for the age characteristics, with the same value of *p* = 0.01.

**Figure 5:**
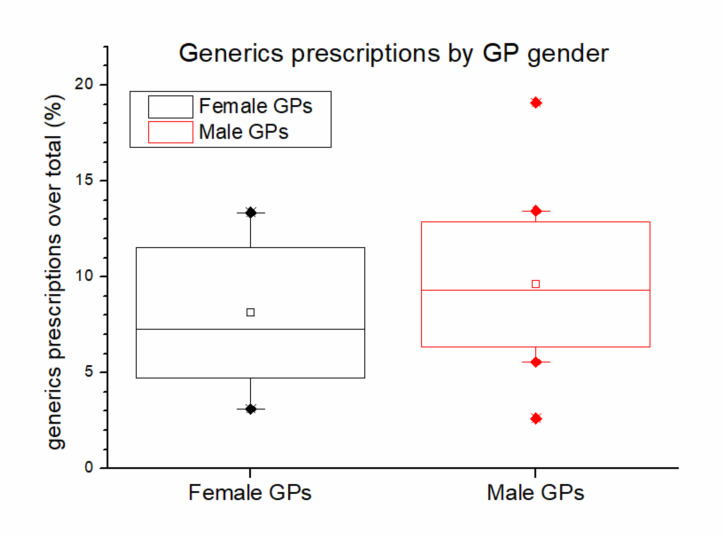
Boxplot of GPs generics prescriptions by GPs gender.

The gender difference in generic drug prescription is presented in Figure 5. In the boxplot, the center line represents the mean, the box represents the standard error for lower and higher limits, and the lines represent the lower and higher limits.

Results from Figure 5 indicate that female GPs have a tendency to prescribe less generics, and this is confirmed by two statistical tests. An unpaired *t*-test shows Male mean = 1627 and Female mean = 1114, with the statistical values of 1.694 and 0.049 respectively, indicating the presence of a significant lower generic drug prescription of female GPs.

**Figure 6:**
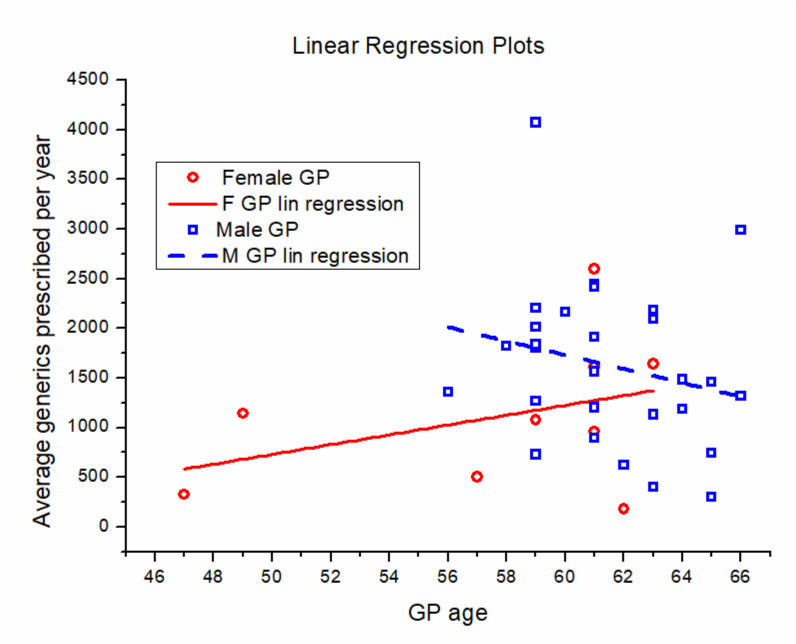
Correlation between average number of generics prescribed per year and GP age (females: red circles; males: blue squares). Full red line: linear regression for females. Dashed blue line: linear regression for males.

In a linear regression analysis, the graph comparison suggests the presence of a gender-related difference in GPs generics prescription behaviors, even if the *p*-values are not significant due to the low dimensions of the samples. Accordingly, we observe an increase in the number of prescribed generics following an increase in the GPs age for females, while we observe the opposite for males, as shown in Figure 6.

Table 4 shows the main groups of the ICD-9-CM classification. The prescriptions of generic drugs have been reclassified accordingly, and the results are presented in the following figures.

**Figure 7:**
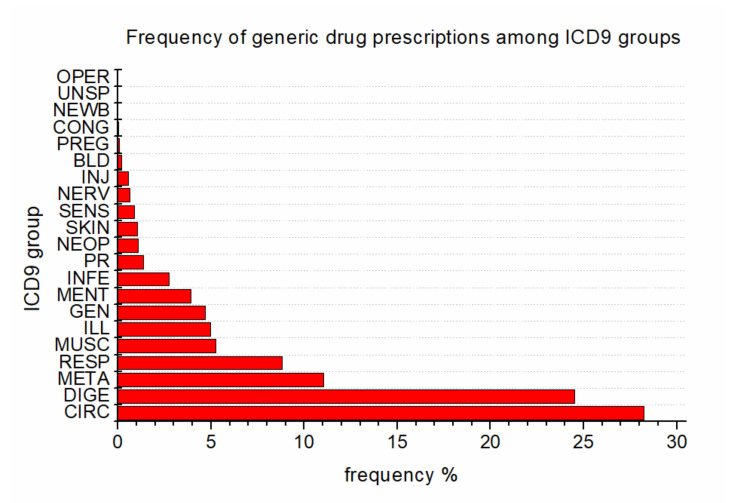
Distribution of generic drugs among the ICD9 groups.

Figure 7 represents the distribution of prescribed generic drugs among the ICD9 groups, that is, the relative frequency of generic drug prescription in each ICD9 group. The values reported may reflect the differences in availability of generic drugs and depend also on the policies of the drug producers. The two most prescribed groups are for the circulatory (28%) and digestive (24%) diseases.

**Figure 8:**
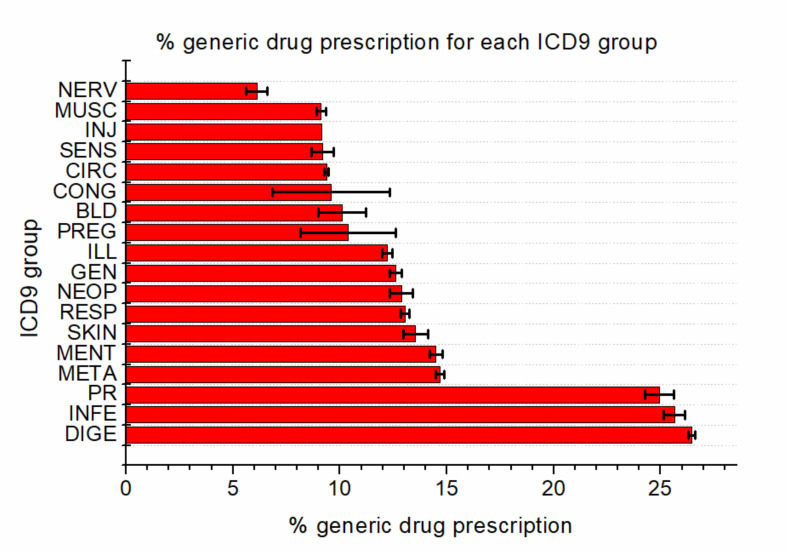
Percentage of generic drug prescriptions within each ICD9 group, with associated statistical uncertainty.

Conversely, in Figure 8, we show the percentage of prescribed generic drugs within each ICD9 group, with the associated statistical error. The data represent the percentage of generic drug prescription with respect to total prescription in each ICD9 group.

The percentage values appear to cluster within a couple of groups: The first one is composed of infections, digestive disease and external causes, with values of around 25% and a very small uncertainty. The second cluster is made up of almost all the other ICD9 groups, with a percentage ranging from 5% to 15%. Only the newborn group shows a very small value and large uncertainty, due to the very small number of prescriptions by GPs, which is understandable as newborns are usually assisted by pediatricians.

## 4. Discussion

The main findings reported in the paper are summarized as follows: All the GPs prescribe generic drugs with an increasing trend; there is a small gender-related difference in prescription behaviors, as female GPs slightly tend to prescribe less generics than male GPs; and finally, in terms of diagnoses, generic drug prescription is concentrated into a specific area, mainly composed of diseases of the circulatory and digestive systems.

A relevant implication is that the generic drug choice is diagnosis-oriented, supporting the assertion implying that, “For several product categories, individuals are likely to show their preferences at the consequence and values levels rather than at the attribute level” [14]. The results support Reynolds and Gutman’s assumption stating that it would be possible to categorize consumers with respect to their personal values for a product category or brand name [14]. Indeed, we should take into account that in the specific case of the drug prescription, there are two important factors: the GPs’ choices mainly in medical/regulatory terms, and the patients’ choices in personal/psychosocial terms. Our study does not investigate the interaction between the patient and the GP in terms of generic drug prescription, and this topic should be taken into account for further investigations.

Our study found that almost all GPs prescribe both generic and brand-name types of drugs to their patients, with differences that may be attributed to the availability of generic drugs, normative indications, GP age, gender and professional experience [15]. The use of EHR (electronic health records) produced for administrative purposes by GPs appears to be a useful basis to study prescription-related information, as already shown by previous studies [16,17] performed on GPs’ EHR databases.

Very few of GPs’ decisions about prescribing generic and/or brand-name drugs can be explained by the observable characteristics which we have been able to access, but all of the evidence indicates that physicians are the key decision-making agents in determining whether patients receive either brand-name or generic drugs, even if there have been suggested other relevant domains [18] that influence generic drug utilization, including patient-related factors, policies and norms, various promotions, education level and technological/scientific advancements.

Our study has two main limitations: The data are of administrative origin, and thus, their epidemiological and healthcare-related significance must be properly inferred, and the number of studied GPs is limited to 38, and it is possible to gain a better statistical significance as the number of GPs significantly increases. This, in principle, is not a fundamental problem, as the National Healthcare System is in possession of the data related to all GPs: their availability should be, in principle, granted to researchers.

## 5. Conclusions

As a conclusion, we can affirm that the prescription of generic drugs, with all its regulatory obligations, also reflects the requests and preferences of the patients. However, we had no possibility to obtain data about the preferences of the patients. The adherence of GPs to regulatory obligations is constant, even with the differences in prescription behavior according to age, gender and experience characteristics of the GPs. The generics prescription also depends on the different diagnoses, and additionally, the market offer of generic drugs is larger where the volume of drugs prescribed is high, and/or the number of patent-expired active principles is high.

This topic needs further research, as many questions remain unclear: On one side, patients appear [19] to be sensitive to their physician’s recommendations, but on the other side, the direct contact between pharmacist and patient/consumer [20] appears to play a significant role for generics promotion. Moreover, as about 60% of the generic drug prescription studies published so far have been [21] directed to cardiovascular, anti-infective and neurological drugs, the other specialty areas have received little attention from scholars. Thus, further research should consider the whole drug therapy process, including GP prescription behaviors, and expand the study area to all generic drugs for different specialty areas. In detail, a survey through GPs could be a useful instrument to know how many patients ask for generic prescription, and it may also be useful to obtain data about feedback from patients on regulatory imposition.

Moreover, a deeper analysis using a larger database at a more granular level of ICD diagnosis could allow us to better understand the interplay between diagnoses and doctor-patient relationship in terms of generic drug prescriptions.

In our knowledge, a limited number of studies have been performed in the Italian setting, and all have focused on a specific area, such as pediatrics [22], antiretroviral therapy [23], five specific high prescription frequency drugs [24] and cardiovascular drugs [25]. Our study is the first to address this topic from a general point of view, using administrative data from GPs’ daily practice.

With these considerations in mind, variation among prescribing decisions is now more understandable. These findings, even if limited, may have important implications for the implementation of evidence-based medicine, which requires a multifaceted approach. Studying the evolution of physician behavior and how it is affected both by mechanisms of information diffusion and by the structure of the healthcare delivery system is an important area for future research.

## Figures and Tables

**Table 1:** Generic drug prescription of female GPs (*n* = 9).

F GP nr.	F GP Age	ANGP	F Gen Yrs
1	47	324.3	6
2	49	1140.8	4
3	57	500.5	6
4	59	1.074.4	7
5	61	1.609.0	4
6	61	2599.5	8
7	61	960.6	13
8	62	180.3	11
9	63	1642.0	4
mean	57.8	1114.6	7.0

**Table 2:** Generic drug prescription of male GPs (*n* = 29).

M GP nr.	M GP Age	ANGP	M Gen Yrs
1	56	1356.1	7
2	58	1821.0	7
3	59	1807.4	7
4	59	1265.7	11
5	59	4075.5	2
6	59	1838.8	6
7	59	2013.7	7
8	59	2204.1	7
9	59	729.4	10
10	60	2166.9	7
11	61	1914.9	7
12	61	1566.0	2
13	61	2446.4	5
14	61	897.4	13
15	61	2412.9	10
16	61	1566.0	2
17	61	1201.7	12
18	62	624.8	13
19	63	1131.9	7
20	63	2091.9	7
21	63	400.1	12
22	63	2187.7	7
23	64	1483.1	7
24	64	1184.3	7
25	65	300.4	5
26	65	1456.4	7
27	65	743.0	3
28	66	1316.7	9
29	66	2989.0	4
mean	61.5	1627.4	7.2

**Table 3:** Descriptive statistics for generic drug prescription by GPs gender.

Variable	F GP Age	F Generics	F Gen Yrs	M GP Age	M Generics	M Gen Yrs
Mean	57.78	1,114.60	7.00	61.48	1627.35	7.24
Standard deviation	5.83	760.60	3.20	2.60	802.01	3.09
Standard error	1.94	253.53	1.07	0.48	148.93	0.57
Upper 95% CL of mean	62.26	1,699.25	9.46	62.47	1932.42	8.42
Lower 95% CL of mean	53.30	529.95	4.54	60.49	1322.28	6.07

Legend: generics = mean generics prescription per year; Gen Yrs = number of years in which a generics prescription is present in the data base.

**Table 4:** ICD-9-CM group codes and description.

Code	Description
INFE	infectious and parasitic diseases
NEOP	neoplasms
META	endocrine nutritional and metabolic diseases and immunity disorders
BLD	diseases of the blood and blood forming organs
MENT	mental disorders
NERV	diseases of the nervous system
SENS	diseases of the sense organs
CIRC	diseases of the circulatory system
RESP	diseases of the respiratory system
DIGE	diseases of the digestive system
GEN	diseases of the genitourinary system
PREG	complications of pregnancy childbirth and the puerperium
SKIN	diseases of the skin and subcutaneous tissue
MUSC	diseases of the musculoskeletal system and connective tissue
CONG	congenital anomalies
NEWB	certain conditions originating in the perinatal period
ILL	symptoms signs and ill-defined conditions
INJ	injury and poisoning
PR	surgical diagnostic and therapeutic procedures

## References

[1] Eric S. (1997). Crisis and learning: A conceptual balance sheet. J. Contingencies Crisis Manag..

[2] Smith D.G. (1993). The effects of copayments and generic substitution on the use and costs of prescription drugs. Inquiry.

[3] O’Brien B. (1984). Patterns of European Diagnoses and Prescribing.

[4] Hassali M.A., Alrasheedy A.A., McLachlan A., Nguyen T.A., Al-Tamimi S.K., Ibrahim M.I.M., Aljadhey H. (2014). The experiences of implementing generic medicine policy in eight countries: A review and recommendations for a successful promotion of generic medicine use. Saudi Pharm. J..

[5] Hellerstein J.K. (1998). The importance of the physician in the generic versus trade-name prescription decision. Rand J. Econ..

[6] Taylor R.J., Bond C.M. (1991). Change in the established prescribing habits of general practitioners: An analysis of initial prescriptions in general practice. Br. J. Gen. Pract..

[7] Denig P., Witteman C.L.M., Schouten H.W. (2002). Scope and nature of prescribing decisions made by general practitioners. BMJ Qual. Saf..

[8] Bensing J.M., van den Brink-Muinen A., de Bakker D.H. (1993). Gender differences in practice style: A Dutch study of general practitioners. Med. Care.

[9] Denig P., Haaijer-Ruskamp F.M. (1995). Do physicians take cost into account when making prescribing decisions?. Pharmacoeconomics.

[10] Lambert M., Blanchin-Roland S., Le Louedec F., Lepingle A., Gaillardin C. (1997). Genetic analysis of regulatory mutants affecting synthesis of extracellular proteinases in the yeast Yarrowia lipolytica: Identification of a RIM101/pacC homolog. Mol. Cell. Biol..

[11] WHO Collaborating Centre for Drug Statistics Methodology (2010). Guidelines for ATC classification and DDD Assignment 2011.

[12] Repubblica Italiana (2006). Decreto Legislativo 193/2006—Classificazione AIC dei Farmaci.

[13] International Classification of Diseases (ICD). http://www.who.int/classifications/icd/en/.

[14] Reynolds T.J., Gutman J. (1988). Laddering theory, method, analysis, and interpretation. J. Advert. Res..

[15] Rodriguez-Calvillo J.A., Lana A., Cueto A., Markham W.A., López M.L. (2011). Psychosocial factors associated with the prescription of generic drugs. Health Policy.

[16] Cavallo P., Pagano S., De Santis M., Capobianco E. (2018). General practitioners records are epidemiological predictors of comorbidities: An analytical cross-sectional 10-year retrospective study. J. Clin. Med..

[17] Cavallo P., Pagano S., Boccia G., De Caro F., De Santis M., Capunzo M. (2013). Network analysis of drug prescriptions. Pharmacoepidemiol. Drug Saf..

[18] Howard J.N., Harris I., Frank G., Kiptanui Z., Qian J., Hansen R. (2018). Influencers of generic drug utilization: A systematic review. Res. Soc. Adm. Pharm..

[19] Drozdowska A., Hermanowski T. (2016). Predictors of generic substitution: The role of psychological, sociodemographic, and contextual factors. Res. Soc. Adm. Pharm..

[20] Zerbini C., Luceri B., Vergura D.T. (2017). Leveraging consumer’s behaviour to promote generic drugs in Italy. Health Policy.

[21] Lucas-Dominguez R., Vidal-Infer A., Alonso-Arroyo A., Navarro C., Valderrama-Zurián J.C., Aleixandre-Benavent R. (2016). Patterns and trends in scientific research on generic drugs. Clin. Ther..

[22] Fabiano V., Mameli C., Cattaneo D., Delle Fave A., Preziosa A., Mele G., Clementi E., Zuccotti G.V. (2012). Perceptions and patterns of use of generic drugs among Italian Family Pediatricians: First round results of a web survey. Health Policy.

[23] Restelli U., Scolari F., Bonfanti P., Croce D., Rizzardini G. (2015). New Highly Active Antiretroviral drugs and generic drugs for the treatment of HIV infection: A budget impact analysis on the Italian National Health Service (Lombardy Region, Northern Italy). BMC Infect. Dis..

[24] Colombo G.L., Agabiti-Rosei E., Margonato A., Mencacci C., Montecucco C.M., Trevisan R. (2013). Off-Patent generic medicines vs. off-patent brand medicines for six reference drugs: A retrospective claims data study from five local healthcare units in the Lombardy region of Italy. PLoS ONE.

[25] Corrao G., Soranna D., Merlino L., Mancia G. (2014). Similarity between generic and brand-name antihypertensive drugs for primary prevention of cardiovascular disease: Evidence from a large population-based study. Eur. J. Clin. Investig..

